# Establishing a robust radioligand therapy program: A practical approach for North American centers

**DOI:** 10.1002/cam4.6780

**Published:** 2024-01-12

**Authors:** Erik S. Mittra, Rebecca K. S. Wong, Celeste Winters, Adam Brown, Shondra Murley, Hagen Kennecke

**Affiliations:** ^1^ Department of Diagnostic Radiology Oregon Health & Science University Portland Oregon USA; ^2^ Department of Radiation Oncology, Princess Margaret Cancer Centre University of Toronto Toronto Ontario Canada; ^3^ Department of Nuclear Medicine West Tennessee Healthcare Jackson Tennessee USA; ^4^ Providence Cancer Institute Portland Oregon USA

**Keywords:** nuclear medicine, program implementation, radioligand therapy, radiopharmaceuticals, theranostics

## Abstract

Radioligand therapy (RLT) is a targeted approach to treating cancer that has been shown to be safe and effective in a variety of disease states, including gastroenteropancreatic neuroendocrine tumors, lymphoma, and most recently, advanced prostate cancer. In the United States, patient access to this therapy is currently variable. Implementation of new RLT programs and expansion of existing programs are needed to broaden patient access to and standardize the delivery of RLT, especially as new therapies are introduced into clinical practice. Drawing from experience in establishing RLT programs in different settings, we have developed practical recommendations for building and implementing a robust RLT program. In this review, we present our recommendations for minimal requirements and optimal requirements, as well as system considerations, and special issues associated with implementing an RLT program in North American centers.

## Graphical abstract

Radioligand therapy (RLT) is a targeted approach to treating cancer that has been shown to be safe and effective in a variety of disease states, including gastroenteropancreatic neuroendocrine tumors, lymphoma, and most recently, advanced prostate cancer. In this review, we present our recommendations for minimal requirements and optimal requirements, as well as system considerations, and special issues associated with implementing an RLT program in North American centers.

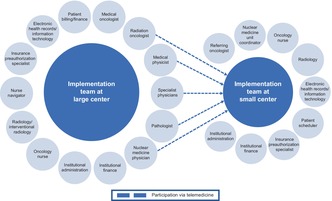

## 1 INTRODUCTION

Radioligand therapy (RLT) uses a ligand that targets cancer cells expressing a specific biomarker combined with a therapeutic radionuclide to deliver cytotoxic radiation.^1^ RLT follows a theranostic approach in which radioligand imaging of a diagnostic biomarker is used to select patients for RLT directed against the same biomarker. Examples of RLTs currently approved by the Food and Drug Administration in the United States are ^90^Y‐ibritumomab tiuxetan used to treat non‐Hodgkin lymphoma (although now rarely used), ^177^Lu‐DOTATATE for the treatment of somatostatin receptor‐positive gastroenteropancreatic neuroendocrine tumors (GEP‐NETs), ^131^I‐iobenguane (high‐specific activity MIBG) for paragangliomas and pheochromocytomas, and, most recently, ^177^Lu‐vivipotide tetraxetan for prostate‐specific membrane antigen‐positive metastatic castration‐resistant prostate cancer.^1^, ^2^, ^3^, ^4^, ^5^, ^6^ Additional targeted therapies utilizing radioligands are in development, with the potential approval of many new agents in the future.

Access to and delivery of these RLTs depend on certain logistical considerations, which vary by the specific RLT modality and institution. The basic infrastructure required to deliver radionuclide therapy includes licensing for radioactive materials, adequate staffing resources, dedicated treatment space, and appropriate imaging equipment.^7^

Despite the proven clinical benefits and relatively modest basic infrastructure requirements of RLT, delivery of and patient access to RLT are variable in the United States.^1^ A large proportion of patients with cancer receive care in smaller community‐based centers with low access to sub‐specialty care^8^, ^9^; these centers may not have enough patient volume to justify the start‐up costs for RLT delivery. Indications for RLT include different cancer types (some rare), which limit the geographic availability of specialists who can advise on therapeutic strategies, contribute to multidisciplinary tumor boards (MTB), and advocate for RLT. Institutional care models for RLT may vary in response to the procedures available at individual institutions and differences in health insurance coverage.^1^

Robust RLT programs are needed to ensure consistent quality of care, especially as new indications and demand for RLT increase. General characteristics of robust RLT programs include efficient treatment delivery that meets and exceeds safety guidelines, scalability in response to clinical demand, and the ability to incorporate evidence‐based care. Robust RLT programs should also be multidisciplinary, facilitating optimal patient selection, care delivery, program growth, and future theranostic practitioner training.

As clinical experience with RLT has grown, organizations have published guidelines for setting up proficient theranostic centers focusing on safety procedures and operational details.^10^ Based on our own extensive clinical experience, we have developed additional general guidance for building and implementing basic and more robust RLT programs. This review describes these practical recommendations and discusses how they vary by context, such as the type of therapy being offered and the clinical setting, with a focus on North American centers.

## 2 METHODS AND GUIDANCE FOR BUILDING A ROBUST RLT PROGRAM

### 2.1 Minimum requirements for implementing a basic RLT program

Basic RLT programs can be implemented in a variety of ways, depending on the context and resources available. Algorithms are available from international bodies (e.g., the European Commission and the International Atomic Energy Agency) to help determine the minimal staffing for a nuclear medicine department to deliver safe, effective, and efficient diagnostic imaging and RLT.^11^, ^12^ Based on our experience, other minimum requirements include the following:

1. A minimum expected caseload to justify the start‐up and ongoing costs of an RLT program (this may vary by institution)
2. A radioactive materials (RAM) license, an authorized user (AU) of radioactive materials, and a radiation safety officer (RSO)
3. Nursing capacity to coordinate and provide supportive care during RLT treatments
4. A nuclear medicine technologist
5. A qualified medical physicist
6. Referring oncologists or other physicians with whom to partner
7. Pharmacy for supportive care medications
8. Appropriate clinical treatment space

#### 2.1.1 Minimum caseload to justify offering radionuclide therapy

Before developing a radionuclide therapy program, a center must consider whether the potential patient volume is adequate to justify implementation. Although there is limited evidence supporting a minimum caseload level, a Society of Nuclear Medicine and Molecular Imaging (SNMMI)‐designated therapy center of excellence must deliver ≥40 administrations/year, and a similar threshold for maintaining competence has been described in Canada.^13^, ^14^ Caseload also affects full‐time equivalents, clinical coordinator time, and other requirements necessary to support the program. Each center also needs to balance patient volume with overall staff, procedure room, and radiopharmaceutical availability to allow appropriate and timely scheduling of treatments. Patient volume beyond minimum thresholds may correlate with better patient outcomes and satisfaction, as it provides the clinical staff with additional experience delivering RLT.^15^, ^16^

#### 2.1.2 Radioactive materials license

Institutions in the United States planning to use nuclear materials for imaging or therapies must obtain a RAM license. This license can be obtained from the state in which the institution resides if that state has an agreement with the Nuclear Regulatory Commission (NRC) to license and regulate certain radioisotopes (i.e., “agreement state”). Currently, 39 states are agreement states.^17^ Institutions in nonagreement states must apply to the NRC directly for a RAM license. Centers may need to make license amendments to offer new types of RLT, for example, to handle alpha‐particle emitters.

#### 2.1.3 Authorized user

To approve a RAM license for the use of radioactive materials in humans, the NRC requires ≥1 AU be named on the license. The AU must be a physician who meets the NRC's training requirements for handling radioactive materials in a medical setting, manages safe material handling, and can supervise others who may need to use radioactive materials for training and other purposes.^18^ The AU is typically a nuclear medicine physician, radiologist, or radiation oncologist. However, the AU can be a physician of any specialty if they have completed all the NRC‐designated training.

#### 2.1.4 Radiation safety program and radiation safety officer

RAM licensing requires an appropriate radiation safety program for the handling and disposal of radioactive materials.^7^, ^10^, ^19^ The RSO is a key participant in this program^18^ and is also responsible for ensuring that institutions meet any additional region‐specific regulations. Although RSOs are ultimately responsible for the safe use of radioactive materials at an institution, the RSO may form a radiation safety team and designate other individuals to perform specific tasks. The RSO (or designee) maintains accurate records of all RLT doses administered at the center, provides radiation safety training to staff and monitors their exposure using dosimeter badges, prepares the treatment room and patient restroom using contamination control measures, provides radiologic surveys as needed, consults with other providers if a patient undergoing RLT has a medical emergency, and helps ensure that the design and structure of new or remodeled facilities meet radiation safety requirements.^18^ The RSO or designee also handles collection, storage, and disposal of radioactive waste. This includes residual or unused doses of radiopharmaceuticals and any contaminated materials generated during therapy. The RSO or designee develops and maintains protocols allowing the safe release of potentially radioactive patients back into the community after treatment.

#### 2.1.5 Nurse

Nurses are critical to the success of an RLT program, with integral roles including patient communication, scheduling, orientation and education, facilitating treatment and concomitant medication administration, monitoring safety, discharging patients, and facilitating MTBs. Cancer infusion room nurses are experienced in the safe handling and administration of complex cancer therapeutics. Nurse navigators are crucial to providing patient education and helping coordinate multidisciplinary RLT care. In some centers, both therapy room nurses and outpatient clinic nurses participate in the RLT process by managing different workstreams. Therapy room nurses focus on RLT delivery and patient discharge, whereas nurses in the outpatient clinic handle pre‐therapy and post‐discharge events. Diverse backgrounds are conducive to nursing roles in an RLT program such as oncology, radiology, and emergency medicine.

#### 2.1.6 Technologist

Technologists ensure that radiopharmaceuticals are received on time and in proper condition for administration.^7^ Technologists should undergo training appropriate for the RLT type they will be handling before beginning work.^7^ Trained technologists are often responsible for ordering radionuclide therapies from manufacturers and receiving, documenting, and inspecting doses upon arrival. The technologist prepares RLT doses, obtains intravenous access, administers the RLT while being vigilant for extravasation, performs radiological surveys of the treatment areas, and, upon treatment conclusion, ensures that the patient radiation level is below the threshold established by the RSO. The technologist also upholds radiation safety protocols set by the RSO in compliance with federal and local regulations.

In smaller centers, nuclear medicine technologists may assume some of the roles that nurses typically fulfill in larger centers such as facilitation of patient arrival and discharge procedures, patient monitoring, and education. For centers where radiation oncology supports care delivery, the technologist is critical to coordinating care within the nuclear medicine department.

#### 2.1.7 Medical physicist

Across RLTs, medical physicists are responsible for accreditation and quality control programs of nuclear medicine and PET equipment. As such, they are involved in designing and reviewing the quality control program, and testing of equipment used for nuclear medicine imaging and therapy. Medical physicists can design structural radiation shielding as “qualified experts.” In cases where dosimetry is performed, the medical physicist develops patient‐specific treatment plans based on radiation dose estimates and biodistribution assessments. Additionally, medical physicists may act as RSOs.

#### 2.1.8 Pharmacy

Pharmacists develop electronic treatment orders and formulary review of supportive care medications. They provide all concomitant medications required on treatment day and flag any concerning medication interactions to minimize this risk during treatment.

#### 2.1.9 Oncologists or other providers with whom to partner

Partner physicians are essential for ensuring appropriate referral for RLT and prevention of interference with other planned treatments (e.g., chemotherapy). They also help assess treatment tolerance, manage toxicities, and assess response to therapy. The partner physician can be a medical oncologist, but many centers also partner with radiation oncologists, surgical oncologists, and other clinical specialists, depending on the cancer type.

#### 2.1.10 Dedicated space and treatment room setup

Physical space requirements include space to receive, store, and prepare radiopharmaceuticals, as well as dedicated treatment rooms and restrooms safely separated from other patients, staff, and general public.^7^

A “hot lab” in which radiopharmaceuticals can be stored and manipulated safely before infusion is needed. It is a restricted‐access room where radiopharmaceuticals are received and confirmed using a dose calibrator. It may also contain personal protective equipment, radiation shields, and radiation survey meters, used during handling of radiopharmaceuticals.

**FIGURE 1:**
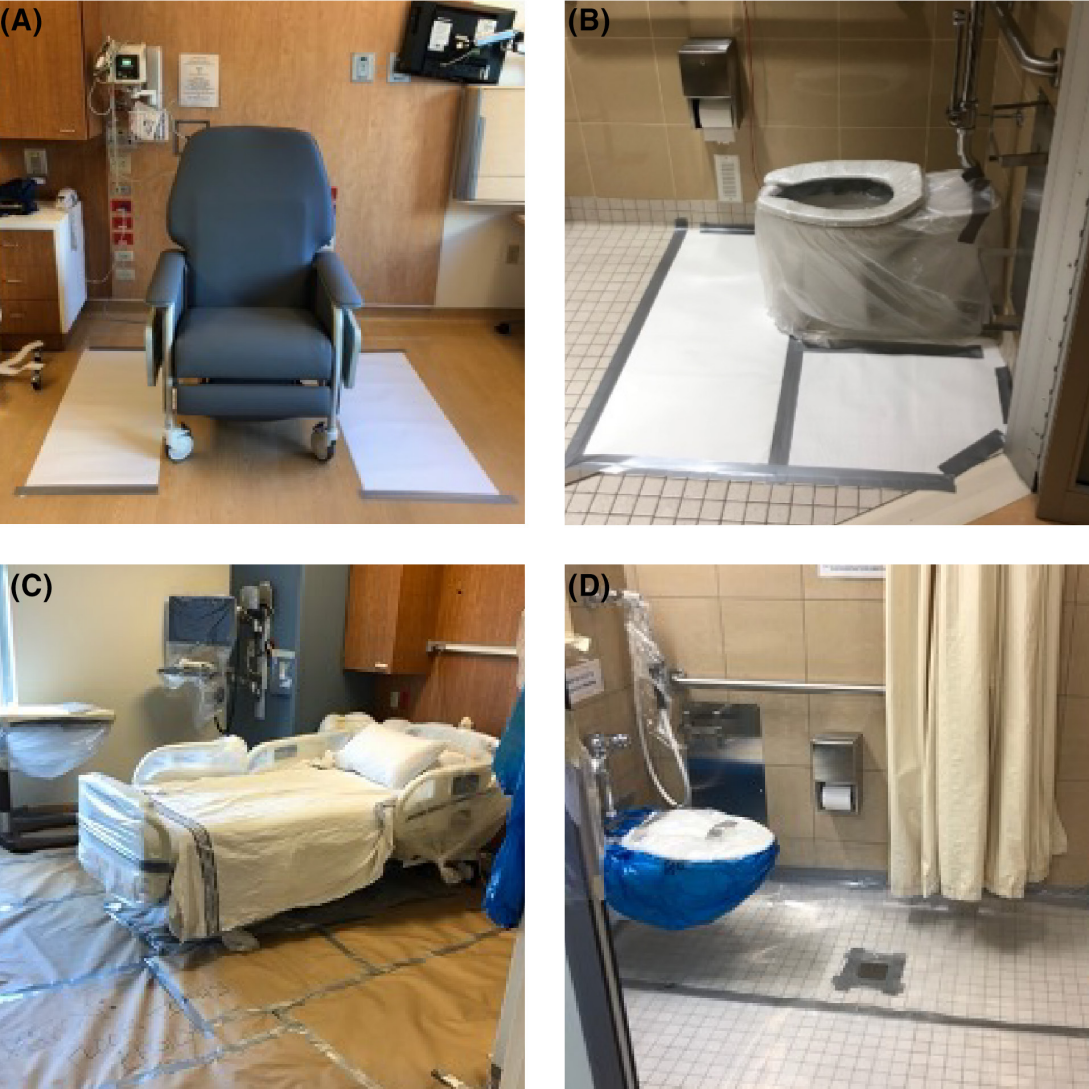
Therapy rooms and dedicated restrooms for patients receiving ^177^Lu‐DOTATATE– and ^131^I‐MIBG. (A) Treatment room for patients receiving ^177^Lu‐DOTATATE. The room itself does not need to be shielded. (B) Dedicated restroom for patients receiving ^177^Lu‐DOTATATE. (C) Treatment room for patients receiving ^131^I‐MIBG. The room is appropriately shielded (lead‐lined). (D) Dedicated, shielded restroom for patients receiving ^131^I‐MIBG.

An appropriate treatment room and dedicated patient restroom are required to keep the radiation exposure to hospital staff and patients as low as reasonably achievable and to maintain adherence to all applicable radiation safety regulations (Figure 1). These can be shared spaces that do not need to be lead‐lined for most outpatient therapies. As this is a high radiation‐contamination area, these restrooms should be designed for easy cleaning.

These spaces do not necessarily need to be within the nuclear medicine department. Spaces well‐suited for delivery of intravenous solutions (e.g., chemotherapy infusion center, selected rooms within an inpatient unit) can be used provided there are proper setup protocols, appropriate communication methods with the RLT team (including after hours), and transfer procedures to an inpatient unit, if necessary. Additional specific features may also be needed depending on the radioisotope used and type of RLT administered (Supplementary Information).

### 2.2 Systems considerations for robust RLT program implementation

Implementing the previously described requirements allows a basic RLT program to be created and sustained. Furthermore, our experience demonstrates that other considerations are important during the evolution of a program from basic to robust. These include the following:

1. Considerations based on treatment type and center size
2. Steering committee to coordinate new RLT program establishment
3. Establish procedures for patient referral, selection, and care coordination
4. Implementation of a multidisciplinary team (MDT)
5. Definition of treatment administration and medication management procedures, including all equipment and administration protocols
6. Outpatient versus inpatient treatment
7. Decontamination and waste disposal procedures
8. Define a patient treatment and follow‐up strategy
9. Patient support considerations—transitioning from clinical trial settings to real‐world settings
10. Establish procedures for preauthorization and billing
11. Consideration of dosimetry approaches

#### 2.2.1 Considerations based on treatment type and center size

Delivery of different RLTs varies in complexity based on the radionuclide used and the radiation safety protocols required. For example, most centers can readily offer radionuclide therapies such as ^223^Ra‐dichloride and ^131^I in the form of sodium iodide, as administration of these agents and subsequent patient monitoring are more straightforward. However, additional infrastructure is needed for other therapies.

Implementation of an RLT program may also differ based on the center size. Large cancer centers often have more resources/staff with expertise in RLT and radiation safety than do smaller community centers. Smaller centers and new adopters of RLT may have less experience navigating the licensing and regulatory nuclear medicine processes. Careful consideration of near‐ and intermediate‐term volume projections is also critical in planning a robust RLT program that is prepared for the future.

#### 2.2.2 Steering committee to coordinate new RLT program establishment

A steering committee composed of key stakeholders should be considered during the early development of the RLT program, helping guide and ensure that the program efficiently meets its objectives. This committee should include members from preauthorization and billing, radiation safety, nuclear medicine, medical oncology, nursing, the pharmacy and formulary team, and other stakeholders involved in the setup and day‐to‐day program operations. Additionally, departmental and institutional leadership and representatives from institutional finance should be included to accurately define the institution's economic impact.

#### 2.2.3 Establish procedures for patient referral, selection, and care coordination

Establishment of referral RLT pathways will ensure appropriate access to therapy. Recognizing the importance of the referral process, the American Society for Radiation Oncology created a framework for developing referral pathways for radionuclide therapy.^20^ This framework highlights the need for physicians involved in the day‐to‐day care of cancer patients (i.e., medical oncologists and primary care providers) to refer potential candidates for RLT to appropriate RLT experts (i.e., nuclear medicine physicians or radiation oncologists). Treated patients may be referred back to their medical oncologists or primary care providers for follow‐up, sometimes with parallel follow‐up from the nuclear medicine physician or radiation oncologist.

Systematic checklists may be used to assist with appropriate patient selection and to identify any relevant concerns that would affect safe RLT delivery. Checklists for ^223^Ra and ^177^Lu‐DOTATATE have been published previously,^7^, ^19^, ^21^ highlighting diagnostic imaging as a part of the theranostic approach and a key component of patient selection for RLT. Before almost all radionuclide‐based therapies, appropriate pre‐therapy imaging is recommended to confirm the expression of the target/biomarker. Thus, centers that plan to perform these therapies must ensure that the necessary imaging studies can be performed. For smaller community centers without advanced imaging capabilities, there should be an appropriate referral plan sending patients to larger centers for advanced imaging.

We encourage AUs to consult with patients before therapy to confirm eligibility, review the therapy goals and timelines, review the necessary radiation safety measures, and ensure compliance. Smaller centers may not have adequate nuclear medicine staff to handle these consultations. Alternatively, referral to a medical oncologist can determine whether RLT is appropriate, followed by proceeding with authorization and scheduling with the nuclear medicine department. Other options include referring potential RLT candidates to large nuclear medicine departments and remote consultations with nuclear medicine physicians.^1^

We also recommend that one or a few individuals be designated to coordinate RLT referrals. This can streamline the process and allow triaged patient visits for urgent cases. The entire process from decision‐making to treatment completion is sensitive to patient status changes, patient comprehension of the processes, radiopharmaceutical delivery, and institution scheduling. Gaps along this pathway can result in treatment disruptions, which may have financial implications for the RLT program and patients. Nurses (coordinators, practitioners, and/or navigators) are well‐suited to coordinate RLT referrals.

#### 2.2.4 Implementation of MDT

RLT requires appropriate patient selection and planning. Consequently, treatment delivery can require coordination and communication among multiple specialties. MDTs are important for providing robust and well‐coordinated care, as they allow team members from various specialties to discuss treatment strategies and patient management.^19^, ^22^ RLT guidelines affirm the importance of MDTs in the successful operation of RLT programs.^10^

**FIGURE 2:**
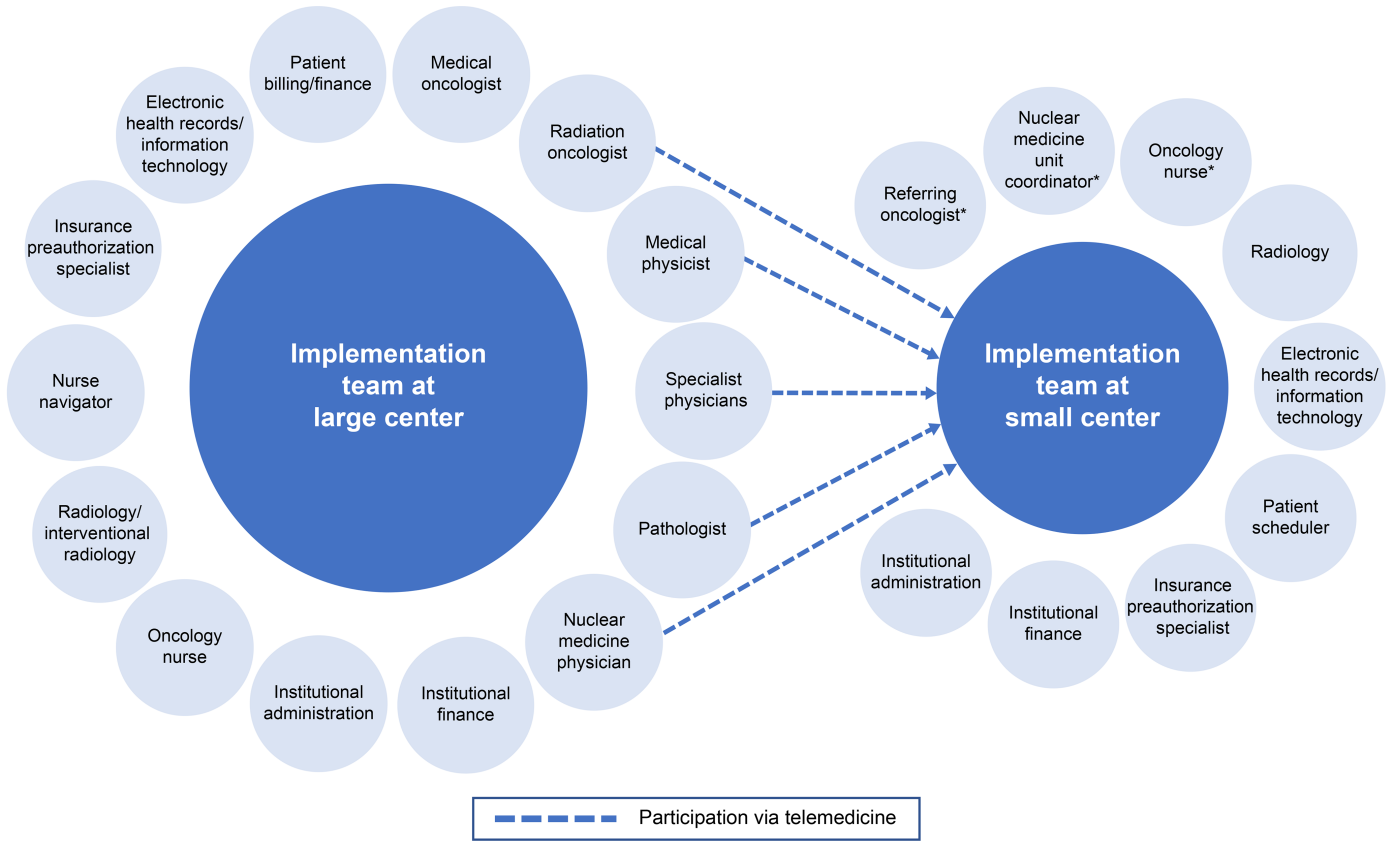
RLT multidisciplinary team. *Denotes a minimum requirement for a basic RLT program. An authorized user and radiation safety officer are also required, and several different types of specialists can fulfill these roles. Possible members of the multidisciplinary implementation and treatment team for large centers (left) and small centers (right). At large centers, members of a multidisciplinary team may include multiple specialists to consult on complex cases. At smaller centers, the team may only consist of the referring oncologist, the oncology nurse, the nuclear medicine unit coordinator, the authorization specialist, and the scheduler. The radiology group can also suggest referral for RLT in scan reports. Telemedicine allows for specialists from large centers to consult at smaller centers.

During the implementation phase of the RLT program, members of the RLT MDT should ideally include the referring physicians, medical oncologists, radiation oncologists, nuclear medicine physicians, nurses, nuclear medicine technologists, radiation safety team members, and pharmacists.^7^, ^19^ If dosimetry is being performed, then qualified medical physicists should be involved in the MDT. Members from electronic health records management, insurance preauthorization, finance/billing, and institutional administration should also be included (Figure 2). It may also be valuable to assign a coordinator to handle scheduling, provide a centralized intake form to capture key patient information, and facilitate recordkeeping of MDT decisions to inform future cases.

Once patient treatment begins, the MDT members may change to reflect ongoing care needs. We suggest regular MDT meetings to ensure potentially eligible patients are reviewed in a timely manner. It is ideal to discuss all cases in the MDT as a new center begins, but eventually this may not be necessary for all patients as consensus decision‐making matures within the group.

Although it may be straightforward to build an MDT that includes members representing specialties such as nuclear medicine and radiation oncology at a large academic center, smaller community centers may not have staff with this expertise. In our experience, an MDT may be built at a smaller center by modifying the different team members to fit the center needs (Figure 2). Smaller centers may also participate in a joint MDT with a larger center.

#### 2.2.5 Definition of treatment administration and medication management procedures, including all equipment and administration protocols

Radionuclide therapy and concomitant medications necessary during therapy should be ordered well in advance. For example, ^177^Lu‐DOTATATE must be ordered from the manufacturer 2 weeks in advance for arrival on a specific date.^7^, ^23^ As supportive medication is standardized for each therapy,^19^, ^22^, ^23^, ^24^, ^25^ an order set can be created and combined with the therapy order to simplify the process. It may be helpful to assign these duties to a nuclear medicine technologist or a nurse coordinator.

Administration of an RLT dose should be performed in accordance with all radiation safety requirements using aseptic techniques and appropriate radiation shielding (Supplemental Information, Supplemental Table 1). The RSO ensures that institutional protocols are being followed via periodic staff training and monitoring.

#### 2.2.6 Outpatient versus inpatient treatment

The decision to administer RLT as an outpatient versus inpatient procedure is determined by the specific radioisotope, the dose, and patient characteristics such as their home situation and other medical issues (Supplemental Table 2). The NRC provides significant guidance, and this is detailed in the Supplemental Information (see “Radiation Safety Guidelines for Patient Charge”). Based on those principles, ^223^Ra dichloride treatment is always performed as an outpatient procedure.^21^, ^26^ ^177^Lu‐based RLT and radioiodine therapy are generally considered outpatient procedures in the United States and Canada, unless a patient has specific comorbidities that increase the likelihood of serious adverse events during therapy^19^, ^25^ or cannot adhere to radiation safety guidelines at home. ^131^I‐MIBG (up to 500 mCi, or 18.5 GBq per cycle) must always be performed as an inpatient procedure due to the high dose. The patient undergoing this procedure is hospitalized for a few days to allow patient radiation levels to come down to a safe level before discharge.^27^, ^28^, ^29^

#### 2.2.7 Decontamination and waste disposal procedures

Proper decontamination and waste disposal procedures are defined in a center's RAM license application to ensure compliance with all federal and state regulations. These regulations ensure that exposure to ionizing radiation is minimized, per the ALARA (as low as reasonably achievable) safety principle, for all individuals involved with the therapy and that radiation levels within an RLT room are reduced below required levels for general public use (a minimum requirement for basic RLT programs). Other waste handling and processing procedures should be specific to the isotopes being used. Radioactive waste is usually stored on site at the center until it has completely decayed (10 half‐lives). At that time, it can be disposed of as either regular waste (if it is not composed of items contaminated with body fluids or chemically reactive/toxic compounds) or hazardous waste (e.g., absorbent paper contaminated with feces).

Another consideration for many centers is the development of policies and procedures for managing post‐discharge complications in radioactive patients. We have found it helpful to define radiation safety recommendations, including clear instructions for emergency scenarios and how to contact the RSO for advice. For example, urinary retention in patients must be handled cautiously after treatment since the urine becomes radioactive. Deceased patients must also be handled using appropriate radiation safety protocols.^10^ We recommend providing 24‐h access to detailed instructions for handling these scenarios to institutions to which the patient could be admitted in the event a complication arises after discharge.

#### 2.2.8 Define a patient treatment and follow‐up strategy

During therapy, providers will need to review patient well‐being, toxicities, laboratory investigations, and any performed imaging. All radionuclide therapies are associated with potential toxicities, with some becoming serious; close follow‐up ensures that these events are identified and managed expeditiously.^19^, ^24^, ^25^ Given that theranostic physicians are not in a position to manage all oncology‐related aspects of patient care, including adverse events, there should be a strong partnership with the referring physician and oncology care team.^7^

The severity and nature of the complication typically dictate who will manage follow‐up (e.g., the nuclear medicine physician or the referring physician). To facilitate consistency, one MDT member should be identified to coordinate patient follow‐up. This individual is often a nurse practitioner or advanced practice practitioner. Alternatively, in smaller community centers this role could be assigned to the referring physician.

Appropriate patient counseling supplemented with patient‐centered educational materials helps patients and caregivers know what to expect during and after RLT. We recommend that patients be broadly counseled on which imaging and laboratory studies will be needed, radiation safety procedures that must be followed during and after therapy, and potential adverse events of therapy at the initial consultation with the nuclear medicine physician (Supplementary Information). After consultation, the nurse, nurse practitioner, physician assistant, and/or the technologist are the key points of contact for subsequent patient education.

#### 2.2.9 Patient support considerations—transitioning from clinical trial settings to “real‐world” settings

It is important to distinguish between RLT implementation in the context of clinical trials versus “real‐world” implementation.^30^ During clinical trials, the clinical trial staff members provide patient support and logistics. However, as centers transition to delivery of RLT as standard of care, institutions will need to ensure adequate administrative and logistical support via nurses and administrative staff.

Patient populations are also different between the two settings. For example, although most clinical investigations with ^177^Lu‐DOTATATE have been conducted in adult patients with advanced, inoperable, well‐differentiated midgut NETs, clinical guidelines include this therapy as a treatment option for other patient groups, including patients with higher‐grade GEP‐NETs and non–GEP‐NET primary neuroendocrine tumors.^31^, ^32^, ^33^ As RLTs gain wider adoption, implementation procedures and guidelines will change as institutions gain experience in additional populations.

#### 2.2.10 Establish procedures for preauthorization and billing

Obtaining insurance preauthorization of RLT allows the staff to focus on the patient. As insurance authorization may take time and delay RLT treatment, key stakeholders who order RLT (i.e., nuclear medicine technologists, nuclear medicine physicians, referring oncologists, and nurses) should be aligned on the process. Information regarding RLT coding and billing, validating authorizations, and providing patient co‐pay assistance is provided by the manufacturer. This information can be shared with all stakeholders at center start‐up to assist in obtaining prompt insurance authorization for RLT.

Some centers recommend that the authorization process be started at the time of the initial referral and consultation with the RLT center^7^, ^19^; however, in our experience, preapproval is generally sought by a managed care team once patient eligibility for RLT is confirmed. We also recommend obtaining authorization for all cycles of therapy beforehand rather than having each cycle of therapy approved individually.

#### 2.2.11 Consideration of dosimetry approaches

The fixed‐dosing approach is commonly used for radiopharmaceuticals, but individualized dosimetry for patients undergoing RLT has been adopted by an increasing number of academic centers, and this approach is the subject of active research.^34^, ^35^ Dosimetry has been proposed to have the advantage of maximizing benefit risk to the patient by calculating the maximum tolerated activity in a personalized manner.^34^ In clinical practice, patients are increasingly being introduced to multiple forms of radiotherapeutic interventions. Quantitative information of radiation dose absorbed has value in estimating toxicity in the real world. However, additional data are needed to determine the clinical benefit of dosimetry.^36^

## 3 SUMMARY AND FUTURE PERSPECTIVE

Therapeutic nuclear medicine is a well‐established treatment approach in oncology, and the RLT demand is expected to grow. In the United States, there are unmet needs in terms of access to and standardized delivery of this treatment modality. We have described best practices for implementing robust RLT programs (Table 1). This guidance was developed from our professional experience initiating and participating in RLT programs and may assist institutions interested in setting up or expanding their own programs. In our experience, minimum RLT center requirements include a minimum caseload, an AU for radioactive materials, an RSO, a nurse, a nuclear medicine technologist, a dedicated space for therapy, pharmacy support, and a partnered oncology physician. Although these requirements will largely remain unchanged, other considerations may need to be adapted as a center begins to offer new therapies to increasing numbers of patients. For example, increasing caseloads will necessitate greater emphasis on efficient patient scheduling for treatment and follow‐up appointments.

**TABLE 1:** Summary of recommendations for implementing RLT at US Centers.

**Minimum requirements** Estimate minimum annual caseload to justify offering RLT Obtain institutional radioactive materials license for the specific radioisotopes and total doses of each used Identify authorized user for radioisotopes used in desired RLTs Develop a radiation safety team, including a radiation safety officer Identify and include other key personnel for RLT preparation and administration Nursing staff to help deliver RLT and monitor patients Nuclear medicine technologists to prepare RLTs Pharmacists to coordinate supportive care medications Identify medical oncologists or other providers (radiation oncologists, surgical oncologists, or other specialists depending on tumor type) with whom to partner for RLT referrals and care coordination Identify and prepare the space within the institution for delivery of RLT, including access to patient bathroom
**Systems considerations** Form a steering committee to guide center setup Establish procedures for patient referral, selection, and care coordination Implement a multidisciplinary treatment team Define procedures for treatment administration and medication management, including all equipment and administration protocols Define protocols for when to treat patients on an outpatient basis and when inpatient treatment may be necessary Implement procedures for decontamination and waste management Establish patient counseling procedures (supplemented with patient educational material) and discharge procedures Define a patient follow‐up strategy by the treating physician, referring physician, or both Determine what changes to patient support logistics will be needed when transitioning from clinical trial to “real‐world” settings Establish procedures for efficient preauthorization and billing

Abbreviations: AU, authorized user; RLT, radioligand therapy; US, United States.

Novel RLTs using different radioisotopes may require additional considerations regarding radiation safety and administration protocols. Furthermore, alternative procedures for delivering existing RLTs may drive changes in the recommended implementation steps. For example, if institutions incorporate dosimetry into their RLT programs, implementation procedures change because of the need to schedule patients for additional dosimetry scans and including a medical physicist in the MDT.^35^ Clinical use of dosimetry is still in its early stages, in large part because most current radiopharmaceutical therapies do not require it (see Supplemental Information).^36^ As such, best practices and uniform methods have yet to be formalized. In addition, the adoption of dosimetry into routine clinical care faces a number of challenges such as the potential need for blood and urine sampling, multi‐timepoint scans, quantitative imaging, and specialized analysis software.^35^, ^36^ Furthermore, the true clinical benefit of dosimetry‐based therapies has yet to be proven. In contrast, the use of fixed doses is significantly more convenient and the ease of integration into clinical practice has allowed widespread use.^36^ Further studies are therefore needed to help define the role of dosimetry in future RLT programs, and there is much ongoing research in this field.

The guidance presented here builds on more granular nuclear medicine–focused global considerations provided by organizations such as SNMMI.^10^ They can help foster collaboration among nuclear medicine physicians, administrators, and other non‐nuclear medicine personnel to build successful RLT programs. However, optimal improvement in delivery and access to these therapies ultimately requires alignment of different components of the health system: governance, regulation, reimbursement, workforce planning, healthcare professional and patient awareness, and data collection.^1^ Besides the administration‐ and system‐level steps we have described, increased RLT education, standardized RLT care in clinical guidelines, and real‐world RLT data collection/analysis will further promote access and delivery of RLT.

As access to RLT increases, the definition of success metrics ensuring that quality of care keeps up with quantity of care will become pivotal. Clinical outcomes metrics are complex and must take into consideration factors such as case mix and case complexity when comparing outcomes across institutions. Alternatively, metrics are often based on institutional‐level targets.^7^, ^19^ Metrics such as patient wait time for therapy, patient satisfaction, claims denial rate, and staff‐to‐patient ratio could be useful benchmarks of various aspects of an RLT program's success.

In conclusion, the implementation of new centers and the expansion of existing centers can broaden access to and improve RLT delivery. Although the process can be challenging, with appropriate guidance and planning, it is achievable even for small centers and ensures these treatments become available to those who need it.

## AUTHOR CONTRIBUTIONS

**Erik S. Mittra:** Conceptualization (lead); data curation (equal); writing – original draft (equal); writing – review and editing (equal). **Rebecca Wong:** Conceptualization (equal); data curation (equal); writing – original draft (equal); writing – review and editing (equal). **Celeste Winters:** Data curation (equal); writing – review and editing (equal). **Adam Brown:** Conceptualization (equal); data curation (equal); writing – original draft (equal); writing – review and editing (equal). **Shondra Murley:** Conceptualization (equal); data curation (equal); writing – original draft (equal); writing – review and editing (equal). **Hagen F. Kennecke:** Conceptualization (equal); data curation (equal); writing – original draft (equal); writing – review and editing (equal).

## FUNDING INFORMATION

Novartis Pharmaceuticals Corporation provided funding for medical writing assistance.

## CONFLICT OF INTEREST STATEMENT

Erik S. Mittra reports consulting fees from Amgen, Curium, Novartis, TerSera, and ITM and research funding from Novartis and Nordic Nanovector. Rebecca K.S. Wong reports education grants from Elekta, Celgene, and Bruce Power, a research grant from Ontario Health, and honoraria from Novartis. Hagen Kennecke reports advisory fees from Tersera and Novartis and research funding from Novartis, Exelixis, and Taiho. No other potential conflicts of interest relevant to this article exist.

## Supporting information


**Data S1:** Supporting Information.

## Data Availability

Data sharing is not applicable to this article as no new data were created or analyzed in this manuscript.
