# Supplementary material for: Establishing a robust radioligand therapy program: A practical approach for North American centers
Source: Cancer Med. 2024 Jan 12;13(3):e6780. doi: 10.1002/cam4.6780 (PMC10905220; doi:10.1002/cam4.6780)
Supplement: Supplementary file 1 — Data S1: Supporting Information. [file CAM4-13-e6780-s001.docx]

**SUPPLEMENTAL INFORMATION**

**Specific Guidance on Physical Space Requirements for Various Types of Radioligand Therapy (RLT)**

^223^Ra is an example of a radionuclide therapy that can be delivered with minimal radiation control procedures in place. It can be safely administered as an outpatient therapy in an unshielded “office” setting (*1,2*). However, while ^177^Lu-based RLTs are also considered outpatient therapies in the United States, a treatment room with private restroom is mandatory for the duration of therapy administration (Figure 1) (*3-5*). Coverage of the floor around the treatment chair with absorbent materials is strongly recommended, especially upon program initiation. This can be used selectively as the team gains experience in radiopharmaceutical handling, patient selection, and support procedures to minimize the risk of spillage. For example, adequate use of antiemetics and careful choice of amino acid preparations can reduce the risk of vomiting, which is a source of radioactive contamination during therapy. The separate patient restroom is critical because patients will need to urinate frequently, and their urine becomes radioactive after treatment. The toilet used by these patients becomes a major source of radiological contamination. Both the toilet itself and the floor around the toilet should be covered with disposable, absorbent material that can be disposed of as radioactive waste in the event of contamination. The treatment room and restroom do not need to be dedicated only to patients receiving ^177^Lu-based therapies, but clear protocols on radiation safety, room release criteria, and decontamination procedures are critical if the room is to be released and used for other purposes. Finally, a shielded (lead-lined) inpatient room with en-suite restroom is required for therapy with ^131^I-MIBG due to the γ emissions from the high-dose ^131^I that the patient will receive (Figure 1).

**Suggested Equipment and Materials for Safe Delivery** **of RLT**

The type of equipment and materials needed will vary depending on the type of therapy being given. Patient hydration and any required premedications or concomitant medications should be available at the time of therapy in addition to the RLT vial itself, sterile sodium chloride infusion bags, sterile intravenous tubing sets with clamps, syringes, intravenous infusion pumps, and syringe pumps. A dose calibrator is required to confirm the activity of the delivered dose. Safety equipment may include tongs for handling radioactive vials, barrier shields, syringe shields, radioactive material spill kits, disinfectant, and a Geiger counter.

**Additional Information Regarding Administration of RLT**

*Dose selection and preparation.* Currently, fixed dosing or empirical dosing selection are the most common dosing approaches used for radionuclide therapies. Often, dosimetry is not required per product labels (*6*), as with ^177^Lu-based RLTs. An exception to this is ^131^I-MIBG, which requires accurate internal pre-therapy dosimetry for the patient and is performed as a two-step process involving the infusion of a small dosimetric dose of ^131^I-MIBG followed by the administration of a therapeutic dose several days to a week later. This process requires a qualified medical physicist. For other therapies, the use of fixed or empirical dosing versus dosimetry for treatment planning varies by institution. In terms of dose preparation, ^177^Lu- and ^131^I-MIBG-based RLTs should be prepared by nuclear medicine technologists using appropriate PPE and shielding (Supplementary Table 1). In contrast, ^223^Ra dichloride can be handled while wearing only latex gloves, and no special shielding is required.

*Administration Protocols.* Administration protocols and concomitant medications required vary based on the type of radioligand being used for treatment. The administration of RLTs such as ^177^Lu-DOTATATE and ^131^I-MIBG is more complex than that of ^223^Ra, which can be performed in the office setting. For example, concomitant medications such as antiemetics (for ^177^Lu-DOTATATE and ^131^I-MIBG) and an amino acid infusion (for ^177^Lu-DOTATATE) must be started before RLT administration; centers wishing to offer this type of RLT must keep in mind that these medications as well as saline solution must be available. Depending on the type of RLT, there may be several options for administering the recommended RLT dosage to patients. For example, ^177^Lu-DOTATATE can be administered with three different methods: gravity method, peristaltic pump method, or the syringe pump method. Each of these methods have their own advantages and disadvantages. Centers should choose amongst them based on patient safety, institutional guidelines/best practices, and product labels.

*Patient Monitoring.* Laboratory evaluations, including blood and pregnancy tests, are common requirements for monitoring patients undergoing RLT. These need to be conducted before treatment to ensure patients meet eligibility requirements (Supplementary Table 2). Patients undergoing RLT also require periodic monitoring during treatment to watch for adverse effects of therapy. For example, special attention is required for patients with functional neuroendocrine tumors receiving ^177^Lu-DOTATATE at risk for triggering carcinoid crisis, and patients undergoing treatment with ^131^I-MIBG need to be monitored for increases in blood pressure after administration. Monitoring can be performed by a nurse with supervision from the authorized user. All laboratory and office visits for patient monitoring should be carefully coordinated and scheduled to avoid delays in therapy, as any unmanaged side effects may disrupt subsequent treatment cycles. In all cases, access to urgent and emergency care procedures should be easily available to on-call or after-hours staff who are not routinely part of the RLT care team. Examples include protocols for the management of extravasation, incontinence, catheter complications, and the potential for a carcinoid crisis or hemodynamic instability during RLT for neuroendocrine tumors.

*Patient Education on Adverse Events*. Since radiopharmaceuticals represent an emerging and growing field, general knowledge of its toxicity profile is expected to be low for patients, caregivers and community health care professionals. A proactive patient education strategy should be implemented as part of the clinical program, with materials tailored to patients in terms of reading level and comprehension. These materials may include a list of potential adverse events and their symptoms. Appropriate counseling about symptoms to report after therapy (e.g., fever, bruising) may assist in rapid identification of any adverse effects (e.g., myelosuppression). The patient materials detailing adverse events should contain relevant clinic telephone numbers for reporting symptoms. Many centers use the educational materials provided by the manufacturer of the RLT and this may be an option that is convenient for community centers. The clinical care team can answer any questions the patient may have regarding identifying adverse events after the procedure.

**Radiation Safety Guidelines for Patient Discharge**

*Regulatory considerations*. Release of patients after RLT is established by the US Nuclear Regulatory Commission (NRC, http://www.nrc.gov) Regulatory Guide 8.39 (or its equivalent in other countries) and further implemented by the local RSO. The NRC guidance allows for the release of patients based on either 1) the administered dose, 2) the measured dose rate at 1 meter from the patient, or 3) patient-specific calculations, all of which ensure that others will not be exposed to more than a dose of 5 mSv (0.5 rem). Importantly, the limits vary by isotope and, for many, can be found in Tables 1 and 2 of NRC 8.39 Revision 1 (*7*). For example, for ^131^I (including NaI as well as MIBG), the first two limits are listed as 33 mCi and 0.07 mSv/hr (7 mrem/hr). For ^177^Lu, on the other hand, those specific limits are not included, but the equations for release based on administered dose can be used. Following administration of 200 mCi of ^177^Lu, assuming a 0.25 occupancy factor at the default distance of 1 meter, and an exposure rate constant of 0.181 R/mCi-hr at 1 cm, NRC 8.39 Rev 1 Equation 2, yields an estimated 2.1 mSv total exposure dose to other individuals (*8*). This is well below the dose limit of 5 mSv (0.5 rem), according to NRC guidelines. This allows for the release of any patient treated with a standard dose ^177^Lu. Of note, the US NRC guidelines are periodically updated and the latest version should always be used.

*Patient/Caregiver Education for Radiation Safety After Discharge.* Patients should be counseled that remaining hydrated before, during, and after therapy will assist in keeping the radiation dose as low as possible. In addition, the healthcare team can provide more detail on specific radiation safety guidelines that should be followed to protect the patient’s close contacts at home. The level of radiation safety instruction varies by type of therapy because of the different radioactive properties of the radionuclides used. For example, many radioisotopes are excreted in the urine and feces. Thus, good toilet hygiene practices and adequate patient hydration after therapy should be encouraged (*2,9,10*).

Disposal of radioactive household items after nuclear medicine procedures has triggered radiation alarms at landfills; in some cases, this resulted in a costly search for the cause of the alarm, which was subsequently billed to the patient (*11,12*). Patients who may be incontinent should be given specific information on how to dispose of used incontinence supplies (e.g., pads, diapers) in the weeks following treatment in order to avoid this. They can be instructed to bag waste contaminated with body fluids separately from household trash and hold it for several weeks to allow the radioactivity to decay before placing it in the household trash.

Restrictions on contact with household members and the general public should also be observed. For example, radiation safety instructions for ^177^Lu-based RLT may recommend staying at least 1 meter (3 feet) away from other people for up to 7 days (*9*). The exact time period of contact restrictions depends on local and institutional regulations, and can be modifed with the approval of the RSO. We recommend that the patient be provided with take-home materials detailing the radiation protection protocols that must be followed at home, including information on how long radiation safety precautions should be followed.

Importantly, patients should be counseled on how to handle travel after therapy, particularly if they must pass through ports of entry such as international airports, as radiation detection equipment at these facilities is quite sensitive (*13*). To assist in these situations, patients should be provided with a “travel card” detailing their therapy and stating that they may be radioactive for several weeks after treatment (*13*).

**REFERENCES**

1. Du Y, Carrio I, De Vincentis G, et al. Practical recommendations for radium-223 treatment of metastatic castration-resistant prostate cancer. *Eur J Nucl Med Mol Imaging.* 2017;44:1671–8.
2. Xofigo (radium Ra 223 dichloride injection) [prescribing Information]. Bayer Pharmaceuticals, Inc.; 2019. https://labeling.bayerhealthcare.com/html/products/pi/Xofigo_PI.pdf. Accessed July 27, 2023.
3. Abbott A, Sakellis CG, Andersen E, et al. Guidance on ^177^Lu-DOTATATE peptide receptor radionuclide therapy from the experience of a single nuclear medicine division. *J Nucl Med Technol.* 2018;46:237–44.
4. Burkett BJ, Dundar A, Young JR, et al. How we do it: a multidisciplinary approach to ^177^Lu DOTATATE peptide receptor radionuclide therapy. *Radiology.* 2021;298:261–74.
5. Mittra ES. Neuroendocrine tumor therapy: ^177^Lu-DOTATATE. *AJR Am J Roentgenol.* 2018;211:278–85.
6. Herrmann K, Giovanella L, Santos A, et al. Joint EANM, SNMMI and IAEA enabling guide: how to set up a theranostics centre. *J Nucl Med.* 2022;49:2300–9.
7. Release of patients administered radioactive material: Regulatory Guide 8.39 Revision 1. U.S. Nuclear Regulatory Commission. 2020. https://www.nrc.gov/docs/ML1923/ML19232A081.pdf. Accessed August 30, 2023.
8. Smith DS, Stabin MG. Exposure rate constants and lead shielding values for over 1,100 radionuclides. *Health Phys*. 2012;102:271–91.
9. Love C, Desai NB, Abraham T, Banks KP, Bodei L, Boike T, et al. ACR-ACNM-ASTRO-SNMMI Practice Parameter for Lutetium-177 (Lu-177) DOTATATE Therapy. *Am J Clin Oncol*. 2022;45(6):233-42.
10. Azedra (iobenguane I 131) [prescribing information]. Progenics Pharmaceuticals, Inc., a Lantheus company; 2021. <https://www.azedra.com/content/pdf/full-prescribing-information.pdf>. Accessed July 27, 2023.
11. Marcus CS, Aldrich R. Avoiding solid waste contamination problems from iodine-131 patients. *J Nucl Med*. 1997;38:26n.
12. Siegel JA, Sparks RB. Radioactivity appearing at landfills in household trash of nuclear medicine patients: much ado about nothing? *Health physics*. 2002;82:367–72.
13. Kendi AT, Mailman JA, Naraev BG, Mercer DJ, Underwood JK, Halfdanarson TR. Patient travel concerns after treatment with ^177^Lu-dotatate. *J Nucl Med.* 2020;61:496–7.
14. Dauer LT, Williamson MJ, Humm J, et al. Radiation safety considerations for the use of ^223^RaCl2 DE in men with castration-resistant prostate cancer. *Health Phys.* 2014;106:494–504.
15. Dash A, Pillai MRA, Knapp FF Jr. Production of ^177^Lu for targeted radionuclide therapy: available options. *Nucl Med Mol Imaging.* 2015;49:85–107.
16. Jimenez C, Erwin W, Chasen B. Targeted radionuclide therapy for patients with metastatic pheochromocytoma and paraganglioma: from low-specific-activity to high-specific-activity iodine-131 metaiodobenzylguanidine. *Cancers.* 2019;11:1018.
17. Hope TA, Abbott A, Colucci K, et al. NANETS/SNMMI procedure standard for somatostatin receptor-based peptide receptor radionuclide therapy with 177Lu-DOTATATE. *J Nucl Med*. 2019;60:937–43.
18. Pluvicto (lutetium Lu 177 vipivotide tetraxetan injection) [prescribing information]. Novartis AG; 2022. <https://www.novartis.com/us-en/sites/novartis_us/files/pluvicto_0.pdf>. Accessed July 27, 2023.
19. Jungels C, Karfis I. ^131^I-metaiodobenzylguanidine and peptide receptor radionuclide therapy in pheochromocytoma and paraganglioma. *Curr Opin Oncol.* 2021;33:33–9.

**SUPPLEMENTARY TABLE 1**. Radiation Safety Considerations for Common Radionuclides Used in Radionuclide or Radioligand Therapy

| **Radionuclide** | **Emission type** | **Half-life** | **Energy** | **Range in tissue** | **Safety requirements** |
| --- | --- | --- | --- | --- | --- |
| ^223^Ra (*14*) | α | 11.4 d | 5.77 MeV | <100 mm | **Minimal**   - α-particle is stopped by gloves/clothing - Shielded treatment room not required - Patients require bathroom hygiene and hydration instructions but no contact precautions |
| ^177^Lu (*15,16*) | β/γ | 6.647 d | β E_max_ = 0.497 MeV  γ: 208 keV, 113 keV | β: 0.67 mm  γ: 6 cm | **Moderate**   - PPE and appropriate shielding should be used during dose preparation and delivery - Shielded treatment room not necessary, but patient must use a separate restroom and be kept well separated from others in the center - Patients must follow detailed home-care instructions to limit exposure to others after therapy |
| ^131^I (*16*) | β/γ | 8.0 d | β E_max_ = 0.606 MeV  γ: 364 keV | β: 0.4 mm  γ: 6 cm | **Extensive**   - PPE and lead shielding required for dose preparation and delivery - Lead-lined treatment room required - Patient must be treated as inpatient for approximately 4 d to allow radiation levels to decrease sufficiently after therapy - Patient must follow detailed home care instructions to limit exposure to others after therapy |

^131^I = iodine-131; ^177^Lu = lutetium-177; ^223^Ra = radium-223; d = days; PPE = personal protective equipment.

**SUPPLEMENTARY TABLE 2.** Recommended Procedure Standards for Radionuclide-Based Therapies According to Clinical Guidelines and Product Labels

| **Therapy** | **Patient Selection Criteria for Administration of RLT** | **Dosing Regimen** | **Required Supportive Medications** |
| --- | --- | --- | --- |
| ^223^Ra dichloride (*2*) | - Symptomatic bone metastases confirmed via bone scintigraphy - Minimal visceral metastases (<3 cm) - Platelet count ≥100 × 10^9^ /L* - Hemoglobin ≥10 g/dL* - Absolute neutrophil count ≥1.5 × 10^9^/L* | 55 kBq (1.49 mCi) per kg body weight, given at 4-week intervals for a total of 6 injections | None |
| ^177^Lu-DOTATATE (*4,9,17*) | - Sufficient tumor uptake on SSTR-based imaging (tumor radiotracer uptake > liver radiotracer uptake) - Hemoglobin >8 g/dL - White blood cell count >2000/mm^3^ - Platelet count >75,000/mm^3^ - Estimated glomerular filtration rate >50 mL/min - Total bilirubin ≤3 x ULN - Serum albumin >3.0 g/dL | 7.4 GBq (200 mCi) every 8 weeks for a total of 4 infusions | - Antiemetics - Amino acid infusion containing L-lysine and L-arginine |
| Lutetium Lu 177 vipivotide tetraxtetan (*18*) | - Documented PSMA expression in tumor/metastases via PET/CT with PSMA-11 imaging agent - Complete blood count and comprehensive metabolic panel do not indicate myelosuppression or kidney/liver abnormalities | 7.4 GBq (200 mCi) every 6 weeks for up to 6 infusions | - Antiemetics |
| ^131^I-MIBG (*10,16,19*) | - Documented tumor uptake of MIBG via diagnostic MIBG scan - Platelet count ≥80,000/mcL - Absolute neutrophil count ≥1200/mcL | *For the dosimetric dose:*   - Patients >50 kg: 185 to 222 MBq (5-6 mCi) - Patients ≤50 kg: 3.7 MBq/kg (0.1 mCi/kg)   *For the therapeutic dose:*   - Patients >62.5 kg: 2 doses of 18,500 MBq (500 mCi) given 90 days apart - Patients ≤62.5 kg: 2 doses of 296 MBq/kg (8 mCi/kg) given 90 days apart   *Therapeutic doses should be adjusted based on the results of the dosimetric administration*. | - Thyroid blockade (inorganic iodine) - Antiemetics |

^*^Selection criteria listed are for the first cycle of therapy only; these criteria may change for subsequent cycles (*2*). ^131^I = iodine-131; ^177^Lu = lutetium-177; ^223^Ra = radium-223; Bq = becquerel; Ci = Curie; MIBG = meta-iodobenzylguanidine; RLT = radioligand therapy; PSMA = prostate-specific membrane antigen; SSTR = somatostatin receptor; ULN = upper limit of normal.
